# Effects of lapatinib or trastuzumab, alone and in combination, in human epidermal growth factor receptor 2‐positive breast cancer: a meta‐analysis of randomized controlled trials

**DOI:** 10.1002/cam4.963

**Published:** 2016-11-23

**Authors:** Yong Xin, Wen Wen Guo, Qian Huang, Pei Zhang, Long‐Zhen Zhang, Guan Jiang, Ye Tian

**Affiliations:** ^1^ Department of Radiotherapy and OncologyThe Second Affiliated Hospital of Soochow UniversitySuzhouChina; ^2^ Department of Radiation OncologyAffiliated Hospital of Xuzhou Medical CollegeXuzhou221002China; ^3^ Department of DermatologyAffiliated Hospital of Xuzhou Medical CollegeXuzhou221002China

**Keywords:** Breast cancer, HER2‐positive, lapatinib, trastuzumab

## Abstract

This meta‐analysis compared the efficiency and safety of lapatinib and trastuzumab, alone or in combination, administered with neoadjuvant chemotherapy in patients with human epidermal growth factor receptor 2 (HER2)‐positive breast cancer. For dichotomous variables, the relative risk ratio (RR) and 95% confidence interval (CI) were used to investigate outcome measures: pathological complete response (pCR), neutropenia, diarrhea, dermatologic toxicity, and congestive heart failure (CHF). Eight randomized controlled trials of 2350 participants (837 receiving lapatinib, 913 trastuzumab, and 555 combination therapy) were selected to compare the efficiency and safety of lapatinib to trastuzumab. A significant difference was found between lapatinib and trastuzumab for pCR (RR *= *0.82, 95% CI: 0.73–0.93; *Z* *=* 3.00; *P = *0.003). In six studies, a significant difference was found between trastuzumab and combination therapy for pCR (RR *= *1.33, 95% CI: 1.18–1.50; *Z* *=* 4.70; *P *<* *0.00001), diarrhea (RR *= *14.59, 95% CI: 7.69–27.67; *Z* *=* 8.20; *P *<* *0.00001), and dermatologic toxicity (RR *= *3.10, 95% CI: 1.61–5.96; *Z* *=* 3.39; *P = *0.007), but none was found for neutropenia (RR *= *1.38, 95% CI: 0.82–2.31; *Z* *=* 1.22; *P = *0.22) or CHF (RR *= *0.14, 95% CI: 0.02–1.17; *Z* *=* 1.02; *P = *0.07). Combination therapy compared to trastuzumab alone increases the pCR rate of HER2‐positive breast cancer patients with no additional cardiac events. Trastuzumab, which is still the first‐line therapy in breast cancer, increases the pCR rate more than lapatinib.

## Introduction

Human epidermal growth factor receptor 2 (HER2) is a member of the ErbB family of receptors and is overexpressed in 15–20% of breast cancer. Considered a prognostic and predictive marker of breast cancer, HER2 is highly aggressive and significantly reduces disease‐free and overall survival 1, 2.

Two therapeutic approaches are used to inhibit HER2‐mediated signaling. The first approach is trastuzumab, a humanized monoclonal antibody that blocks the activity of HER2, which improves the prognosis of early and advanced stage HER2‐positive breast cancer 3. The second approach is lapatinib, an orally active small molecule that reversibly inhibits HER1 and HER2, best studied in clinical trials to date. Lapatinib is often administered in combination with endocrine therapy or with capecitabine 4. Trastuzumab offers better pathological complete response (pCR) rates with no additional toxicity when administered with neoadjuvant chemotherapy for the treatment of HER2‐positive breast cancer 5. Preclinical studies of the trastuzumab and lapatinib combination have also suggested that dual targeting is more effective than single‐agent targeting 6. Assessment of the clinical benefit of administering the lapatinib and trastuzumab combination with chemotherapy for operable HER2‐positive breast cancer has proved positive 7, but replacement of lapatinib with trastuzumab as first‐line targeted therapy in breast cancer has demonstrated conflicting results. Neoadjuvant chemotherapy is the standard treatment for women who present with large, locally advanced breast tumors.

A meta‐analysis of all relevant published randomized, controlled trials (RCTs) was performed to compare the efficiency and safety of lapatinib and trastuzumab, alone or in combination, administered with neoadjuvant chemotherapy in patients with HER2‐positive breast cancer.

## Patients and methods

### Search strategy and study selection

The search strategy consisted of a systematic review of the literature for RCTs over the last 5 years in any language in the Wanfang Data, PubMed, the Cochrane Library, Medline, and EBSCO databases. The publication time searched was from March 2011 to March 2016. The search terms used were “trastuzumab,” “lapatinib,” “her‐2 positive,” “breast AND (cancer OR tumor OR carcinoma),” and “randomized controlled trials OR RCT.” Article selection was based on the methodology used in the RCTs. A total of 39 Chinese articles and 506 English articles were identified. These articles were reviewed and screened for duplicate or incomplete data. When relevant data were unclear, the articles were read by different investigators and then discussed.

### Data extraction

Inclusion criteria were RCTs that (1) pathologically confirmed breast cancer and HER2 positivity, (2) patients aged over 18 years, (3) chemotherapy tolerance, (4) expected lifetime of more than 3 months, (5) compared trastuzumab with lapatinib or trastuzumab versus the combination therapy, and (6) reported sufficient data on outcomes. Exclusion criteria were (1) nonrandomized and nonclinical controlled trials, (2) trials with missing data, and (3) duplicate reports, trials of poor methodological quality and trials with obvious bias.

Data extracted from each article included the name of the first author, year of publication, journal name, study quality, intervention, number of patients in the study, dosage used in the three groups (lapatinib, trastuzumab, and combination therapy), and the number of patients with different endpoints.

Several outcomes were measured: pCR, neutropenia, diarrhea, dermatologic toxicity, and congestive heart failure (CHF). pCR was defined as the absence of any invasive component in the resected breast specimen. Adverse events were graded according to the National Cancer Institute Common Toxicity Criteria Version 3.0. The number of patients with grade 3–4 adverse events was determined from the articles. Disagreement regarding data extraction was resolved by discussion and consensus among the investigators.

### Quality assessment

The methodological quality of the RCTs was assed according to the Cochrane Handbook for Systematic Reviews of Interventions Version 5.0.0: (1) random sequence generation, (2) concealment of allocation, (3) blinding of participants and personnel, (4) blinding of the outcome assessment, (5) incomplete outcome data, (6) selective reporting, and (7) other sources of bias.

### Statistical methods

Data were analyzed using Review Manager v.5.3 software (Cochrane Collaboration, Oxford, UK). For dichotomous variables, outcomes were calculated as the relative risk ratio (RR), the 95% confidence interval (CI), and a *P*‐value of <0.05 was considered statistically significant. The inconsistency index (*I* ^2^) statistic and the *Q* statistic were used to test the heterogeneity between RCTs 7. For outcomes with fine homogeneity (*P *>* *0.1; *I* ^2^ ≤ 50%), a fixed effect model was used for secondary analysis; otherwise (*P *<* *0.1; *I* ^2^ > 50%), a random‐effect model was used 7.

## Results

### Included studies

**Figure 1:**
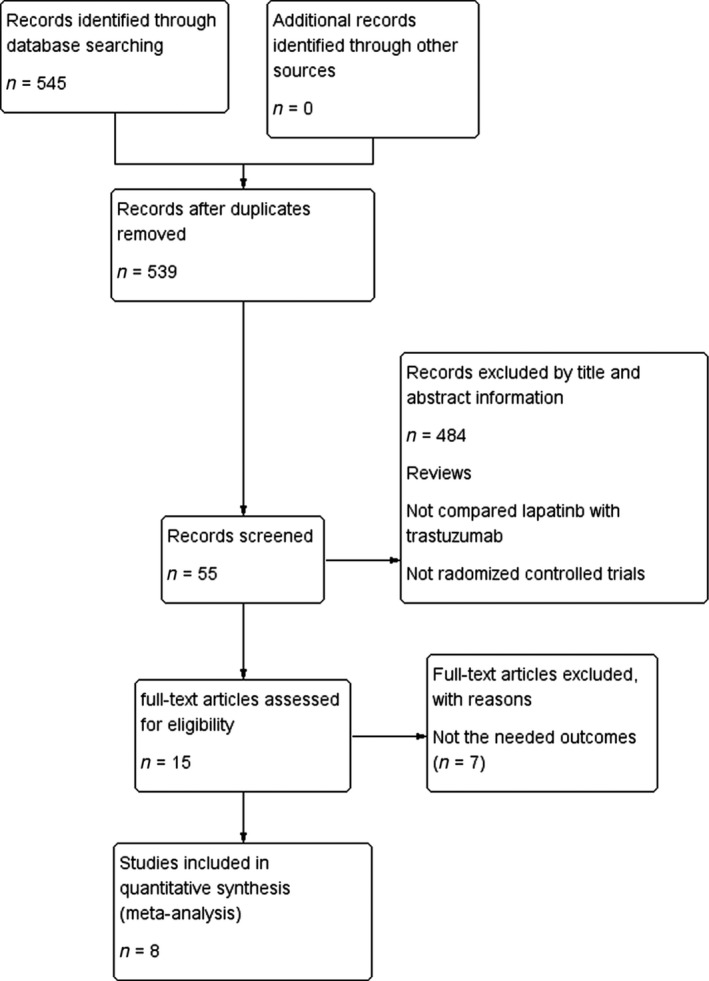
Flowchart of article screening and the selection process.

A total of 39 Chinese papers and 506 English papers were selected, published from March 2011 to March 2016. The literature selection process is presented in the PRISMA flowchart (Fig. 1) according to the PRISMA guidelines. After comprehensive discussion and analysis of the full text, eight RCTs were selected and included in the final meta‐analysis.

The selected RCTs included a total of 2350 patients with pathologically confirmed breast cancer. Of these patients, 837 received lapatinib, 913 received trastuzumab, and 555 received the combination therapy. Each RCT applied different modes of neoadjuvant therapy and different doses of experimental drugs. Table 1 presents the characteristics of the RCTs.

**Table 1:** Characteristics of the eight RCTs (L: lapatinib; T: trastuzumab)

Author	Phase	Groups	No. of patients per group	Neoadjuvant anti‐HER2 therapy	Duration of anti‐HER2 therapy
Bonnefoi H 17	II	L	22	1000mg daily	12weeks
		T	53	4mg/kg → 2mg/kg weekly	12weeks
		L+T	50	L:1000mg daily + T:2mg/kg weekly	12weeks
Baselga J 18	III	L	154	1500mg daily	18weeks
		T	149	4mg/kg → 2mg/kg weekly	18weeks
		L+T	152	L:1000mg daily + T:2mg/kg weekly	18weeks
Untch M 15	III	L	308	1250mg daily	24weeks
		T	307	8mg/kg → 6mg/kg every 3 weeks	24weeks
GuarneriV 3	II	L	38	1500mg daily	26weeks
		T	36	4mg/kg → 2mg/kg weekly	26weeks
		L+T	45	L:1000mg daily + T:2mg/kg weekly	26weeks
Robidoux A 6	III	L	171	1250mg daily	16weeks
		T	177	4mg/kg → 2mg/kg weekly	16weeks
		L+T	171	L:750mg daily + T:2mg/kg weekly	16weeks
Holmes FA 19	II	L	26	1250mg daily	26weeks
		T	29	4mg/kg → 2mg/kg weekly	26weeks
		L+T	23	L:750mg daily + T:2mg/kg weekly	26weeks
Carey L 16	III	L	64	1500mg daily	16weeks
		T	118	4mg/kg → 2mg/kg weekly	16weeks
		L+T	117	L:1000mg daily + T:2mg/kg weekly	16weeks
Alba E 20	II	L	52	1250mg daily	12weeks
		T	50	8mg/kg → 6mg/kg every 3 weeks	12weeks

### Methodological quality

**Figure 2:**
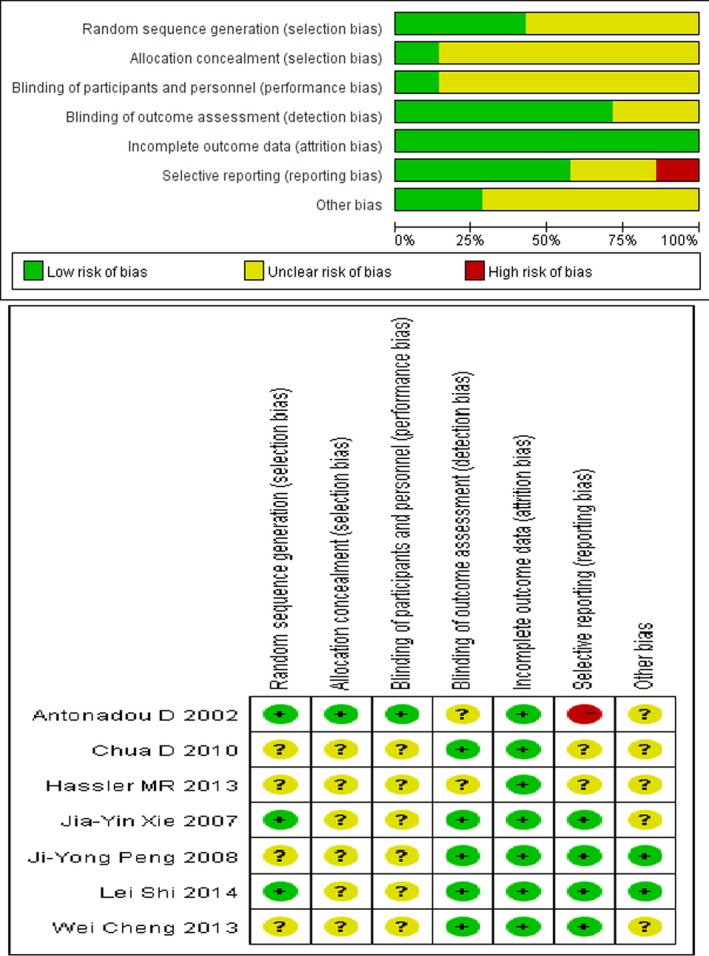
Risk of bias percentile chart.

**Figure 3:**
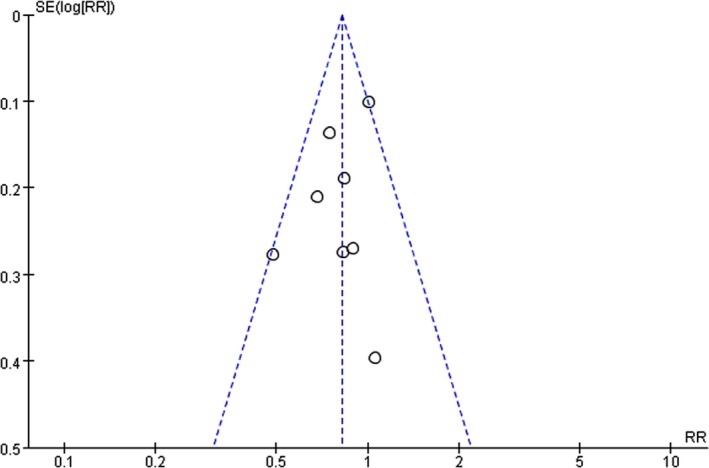
Funnel plot of the publication bias.

Five RCTs referred to restricted randomization methods, such as permuted blocks design or biased‐coin algorithm. Seven RCTs included patients who discontinued treatment because they refused surgery or did not meet the test requirements (Fig. 2). The funnel plot, which was substantially symmetrical, was used to analyze the publication bias (Fig. 3). Considering that the meta‐analysis involves a relatively small number of RCTs, a certain degree of publication bias exists.

### Pathological complete response rate

**Figure 4:**
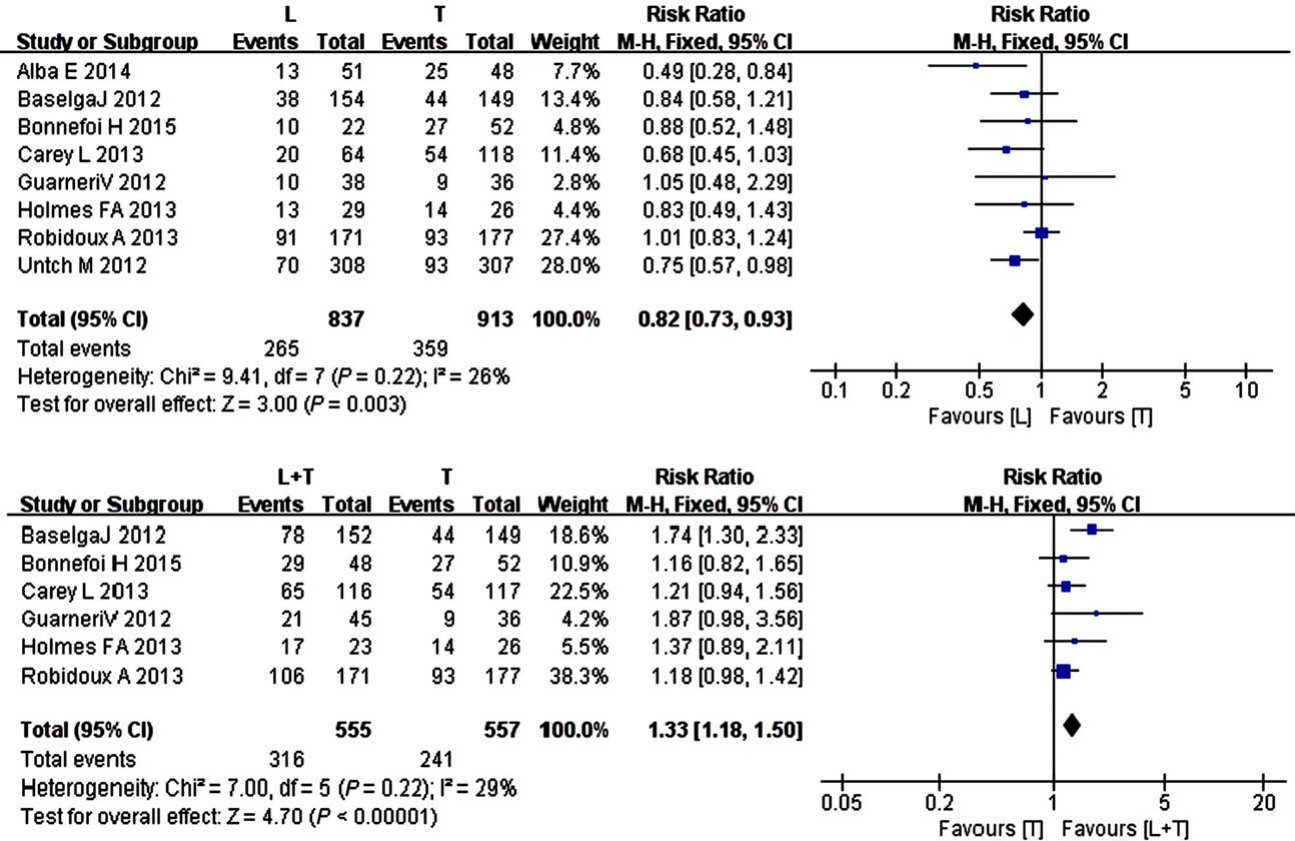
Forest plot of the pCR rate for lapatinib, trastuzumab, and combination therapy.

The pCR rate was analyzed in eight studies including 1750 patients (*n* *=* 837 in the lapatinib group; *n* *=* 913 in the trastuzumab group). The heterogeneity test was not statistically significant; therefore, data for each outcome was calculated using the fixed effects model (*I* ^2^ =* *26%; *P = *0.22). The meta‐analysis indicated a significant difference in the pCR rate in patients treated with trastuzumab compared to lapatinib (RR = 0.82, 95% CI: 0.73–0.93; *Z* *=* 3.00; *P = *0.003) (Fig. 4). Six studies administering combination therapy (*n* *=* 555 in both groups) were also analyzed. Compared to trastuzumab alone, combination therapy showed a higher pCR rate (RR = 1.33, 95% CI: 1.18–1.50; *Z* *=* 4.70; *P *<* *0.00001) (Fig. 4).

### Neutropenia

**Figure 5:**
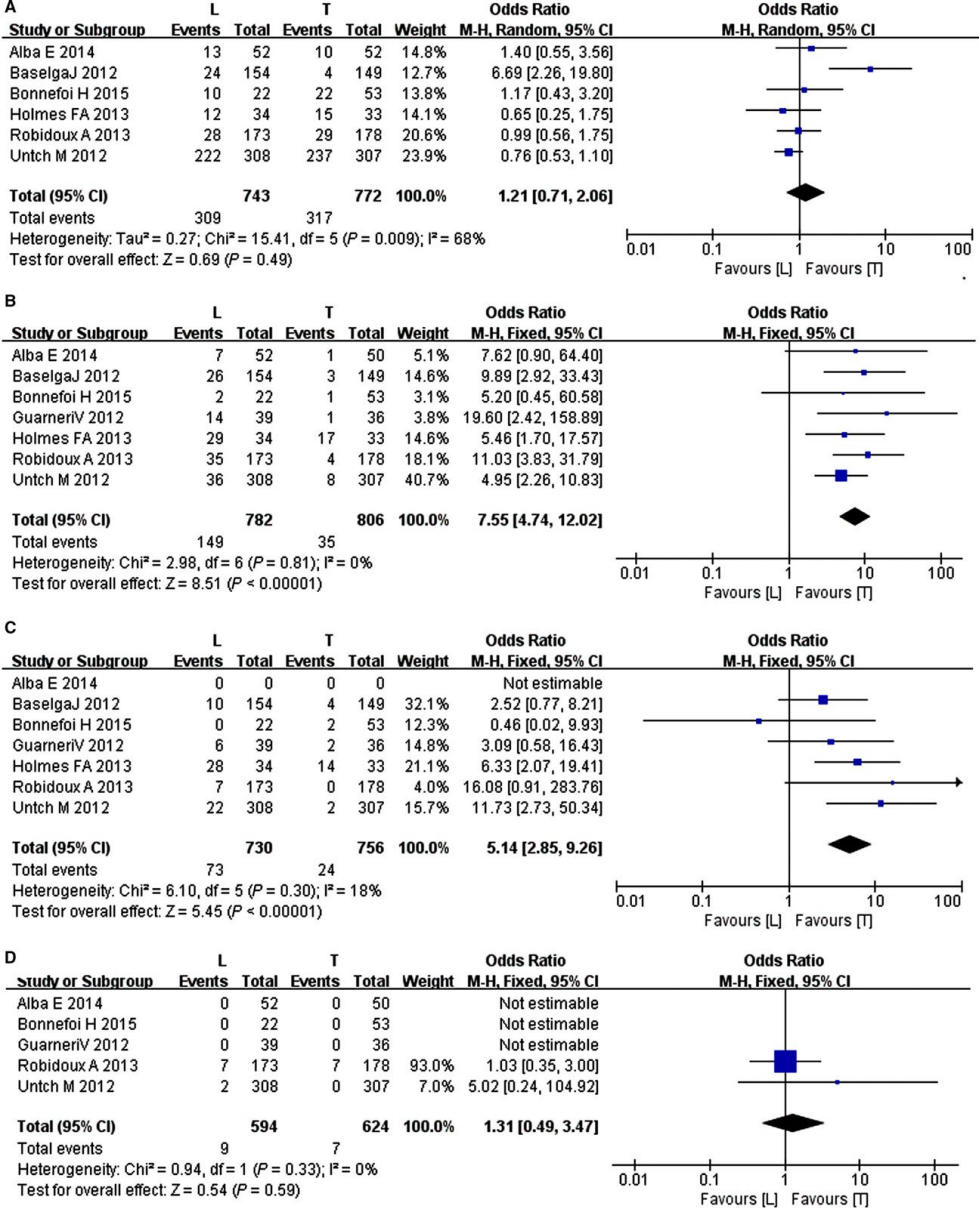
Forest plot of the adverse events for lapatinib and trastuzumab: (A) neutropenia, (B) diarrhea, (C) dermatologic toxicity, (D) congestive heart failure.

The random effects model was used to assess the neutropenia adverse event between the lapatinib and trastuzumab groups as the heterogeneity test was significant (*I* ^2^ *= *68%; *P = *0.009). The six studies included in the meta‐analysis indicated no significant difference in the neutropenia adverse event between the lapatinib and trastuzumab group (RR *= *1.21, 95% CI: 0.71–2.06; *Z* *=* 0.69; *P = *0.49) (Fig. 5).

**Figure 6:**
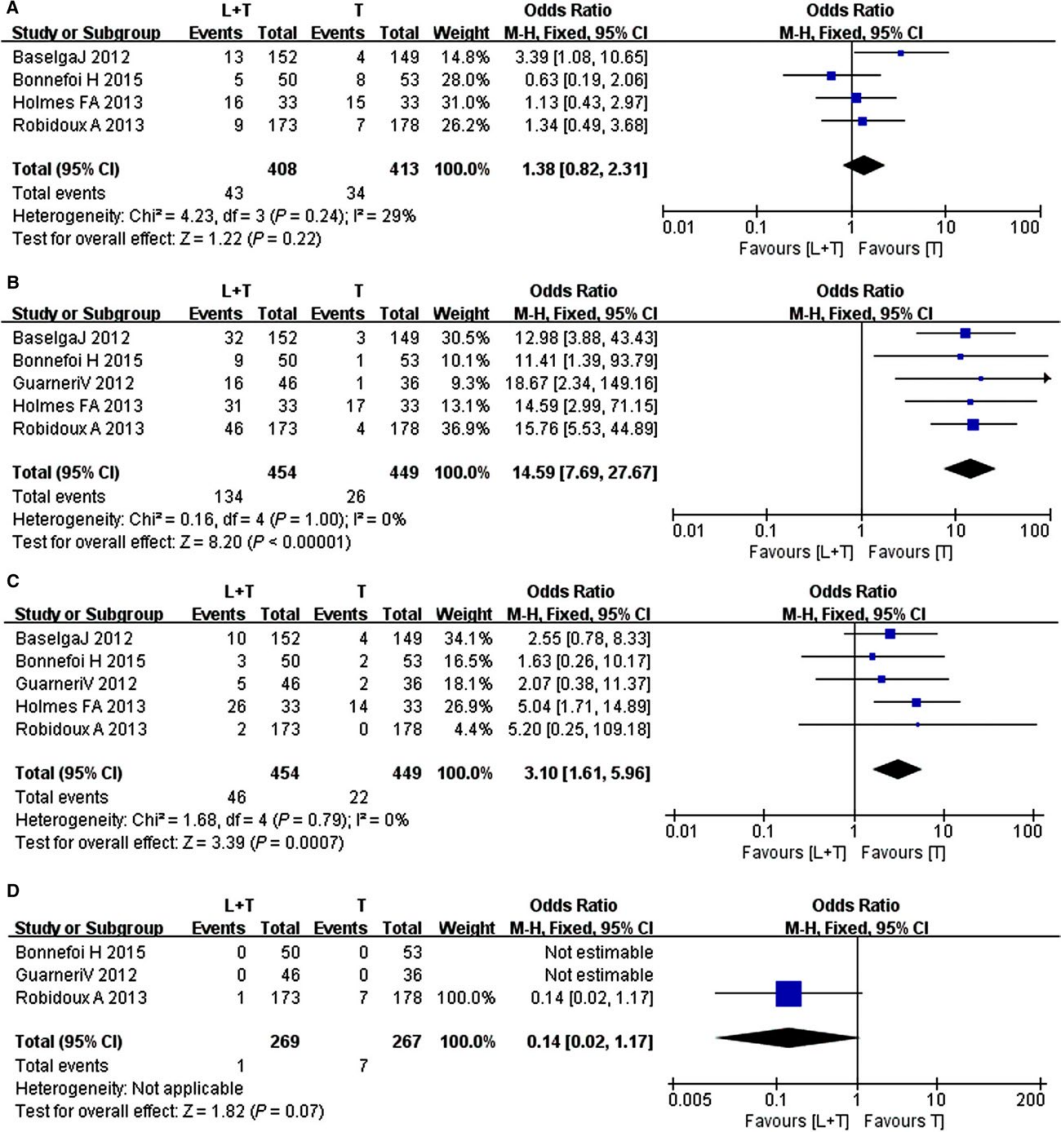
Forest plot of the adverse events for trastuzumab and combination therapy: (A), neutropenia, (B) diarrhea, (C) dermatologic toxicity, (D) congestive heart failure.

The random effects model was also used to assess the neutropenia adverse event between the trastuzumab and the combination groups as the heterogeneity test was significant (*I* ^2^ *= *29%; *P = *0.24). The four studies included in the meta‐analysis indicated no significant difference in the neutropenia adverse event in patients between the trastuzumab and combination groups (RR *= *1.38, 95% CI: 0.82–2.31; *Z* *=* 1.22; *P = *0.22) (Fig. 6).

### Diarrhea

The fixed effects model was used to analyze the diarrhea adverse event between the lapatinib and trastuzumab groups as the heterogeneity test was not significant (*I* ^2^ *= *0%; *P = *0.81). The seven studies included in the meta‐analysis indicated a significant difference in the diarrhea adverse event in patients between the lapatinib and trastuzumab groups (RR *= *7.55, 95% CI: 4.74–12.02; *Z* *=* 8.51; *P *<* *0.00001) (Fig. 5).

The fixed effects model was also used to analyze the diarrhea adverse event between the trastuzumab and combination groups as the heterogeneity test was not significant (*I* ^2^ *= *0%; *P = *1.00). The four studies included in the meta‐analysis indicated a significant difference in the diarrhea adverse event in patients between the trastuzumab and combination groups (RR *= *14.59, 95% CI: 7.69–27.67; *Z* *=* 8.20; *P *<* *0.00001) (Fig. 6).

### Dermatologic toxicity

The fixed effects model was used to analyze the diarrhea adverse event between the lapatinib and trastuzumab groups as the heterogeneity test was not significant (*I* ^2^ *= *18%; *P = *0.30). The seven studies included in the meta‐analysis indicated a significant difference in the dermatologic toxicity in patients between the lapatinib and trastuzumab groups (RR *= *5.14, 95% CI: 2.85–9.26; *Z* *=* 5.45; *P *<* *0.00001) (Fig. 5).

The random effects model was used to analyze the diarrhea adverse event between the trastuzumab and combination groups as the heterogeneity test was not significant (I^2^ *= *0%; *P = *0.79). The five studies included in the meta‐analysis indicated a significant difference in the dermatologic toxicity in patients between the trastuzumab and combination groups (RR *= *3.10, 95% CI: 1.61–5.96; *Z* *=* 3.39; *P = *0.007) (Fig. 6).

### Congestive heart failure

The fixed effects model was used to analyze the diarrhea adverse event between the lapatinib and trastuzumab groups as the heterogeneity test was not significant (*I* ^2^ *= *0%; *P = *0.33). The five studies included in the meta‐analysis indicated no significant difference in the CHF in patients between the lapatinib and trastuzumab groups (RR *= *1.31, 95% CI: 0.49–3.47; *Z* *=* 0.54; *P = *0.59) (Fig. 5).

Only three studies included both the trastuzumab and the combination groups described the outcome, which indicated no significant difference in CHF in patients between the trastuzumab and the combination groups (RR *= *0.14, 95% CI: 0.02–1.17; *Z* *=* 1.02; *P = *0.07) (Fig. 6).

## Discussion

This study compared the efficiency and safety of lapatinib and trastuzumab, alone or in combination, combined with neoadjuvant chemotherapy in patients with HER2‐positive breast cancer. The meta‐analysis provided evidence that lapatinib and trastuzumab combination therapy significantly increased the pCR rate. Trastuzumab increased the pCR rate more than lapatinib. Lapatinib caused skin rash, diarrhea, and other adverse events.

Neoadjuvant chemotherapy has been reported to be equivalent to adjuvant chemotherapy in terms of survival and overall disease progression in the treatment of breast cancer 8. Neoadjuvant therapy is widely used in the treatment of breast cancer and its clinical application offers certain attractive advantages: first, the treatment reduces tumor size and increases breast‐conserving surgery rates; second, it provides an intuitive evaluation for predicting treatment efficacy; third, it evaluates patient prognosis as the pCR rate is a proven reliable prognostic factor 9, 10, 11. Patients that attain a pCR rate during neoadjuvant therapy exhibit a significantly improved disease‐free survival rate 5.

Trastuzumab substantially improves the efficacy of chemotherapy in HER2‐positive breast cancer patients. Resistance to trastuzumab still develops in some women after adjuvant therapy, which increases the importance of the development of additional agents that target HER2 through different mechanisms of action 6. Lapatinib is efficient in patients with HER2‐positive metastatic breast cancer that progressed after trastuzumab treatment. When trastuzumab fails in the target treatment, second‐line therapy with lapatinib is 8% efficient 12. As trastuzumab and lapatinib act on different parts of the HER2 receptor, the therapies are complementary in principle and in mechanism. Preclinical experiments have confirmed that the combination of these two drugs synergistically inhibit breast cancer cell growth and enhance the HER2 blocking effect 13. In addition, comprehensive anti‐HER2 therapy appears to reduce HER2 resistance 14.

In the eight RCTs, the pCR rate as the first endpoint was analyzed for the lapatinib, trastuzumab, and combination groups. A significant difference in the pCR rate between the lapatinib and trastuzumab groups was found (*P = *0.003). Moreover, the pCR rate of the combination group was 1.33 times higher than that of the trastuzumab group. These results might be explained by the lower ability of lapatinib to block the HER2 pathway compared to trastuzumab. Furthermore, trastuzumab may have additional antitumor efficacy through the recruitment of immune effector cells responsible for antibody‐dependent cytotoxicity and inhibiting angiogenesis 1. In the CHER‐LOB 3 and NSABP 6 trials, the pCR rates in the lapatinib group were slightly higher than in the trastuzumab group, which may be explained by the relatively small number of patients analyzed, and the variation in dosage and courses of the targeted drugs among the trials. The results of this study demonstrated that combination therapy is better than trastuzumab alone in increasing the pCR rate.

The meta‐analysis analyzed grade 3–4 adverse events according to the national criteria. Common adverse reactions of lapatinib were diarrhea and skin rash while trastuzumab showed potentially serious cardiac toxicity. The incidence of adverse events, such as diarrhea and skin rash, in the lapatinib group was significantly higher than in the trastuzumab group. Lapatinib and trastuzumab combination therapy also exhibited the same effect. Surprisingly, the results demonstrated that the combination did not increase cardiac toxicity compared to trastuzumab, which indicates that combination therapy is safe and effective.

This meta‐analysis had several limitations. As shown in Table 1, only a limited number of eligible studies and a relatively small number of patients were analyzed. Each study used different drug doses and different neoadjuvant therapy programs. Of the eight English‐language RCTs, five 3, 6, 15, 18, 19 referred to restricted randomization methods, such as permuted blocks design or biased‐coin algorithm, while the other RCTs 16, 17, 20 did not specify the random allocation method. As positive results are more likely to be published, publication bias should also be considered. In addition, eight studies were randomized, open‐label, multicenter trials. Participants and investigators were not blinded to the therapy assignment, but researchers accessing the outcome were blinded to the therapy group. As outcomes including pCR, neutropenia, diarrhea, dermatologic toxicity, and CHF are objective, the lack of blinding of participants and investigators would not have caused significant bias.

In conclusion, the currently available evidence shows that lapatinib and trastuzumab combination therapy significantly increases pCR rates in HER2‐positive breast cancer patients with no additional cardiac side effects compared to trastuzumab alone. Trastuzumab is better than lapatinib according to the pCR rate from our data. However, trastuzumab is still the first‐line targeted therapy in breast cancer and cannot be replaced by lapatinib because of cost considerations. Large, high‐quality, double‐blind trials are needed to confirm the efficiency of lapatinib and trastuzumab combination therapy, and verify whether combination therapy can prolong disease‐free survival or overall survival.

## Conflict of Interest

The authors declare that they have no conflict of interest.
